# Soil microbial community composition and respiration along an experimental precipitation gradient in a semiarid steppe

**DOI:** 10.1038/srep24317

**Published:** 2016-04-14

**Authors:** Cancan Zhao, Yuan Miao, Chengde Yu, Lili Zhu, Feng Wang, Lin Jiang, Dafeng Hui, Shiqiang Wan

**Affiliations:** 1 State Key Laboratory of Cotton Biology, Key Laboratory of Plant Stress Biology, College of Life Sciences, Henan University, Kaifeng, Henan 475004, China; 2 School of Biology, Georgia Institute of Technology, Atlanta, GA 30332, USA; 3 Department of Biological Sciences, Tennessee State University, Nashville, TN 37209, USA

## Abstract

As a primary limiting factor in arid and semiarid regions, precipitation strongly influences soil microbial properties. However, the patterns and mechanisms of soil microbial responses to precipitation have not been well documented. In this study, changes in soil microorganisms along an experimental precipitation gradient with seven levels of precipitation manipulation (i.e., ambient precipitation as a control, and ±20%, ±40%, and ±60% of ambient precipitation) were explored in a semiarid temperate steppe in northern China. Soil microbial biomass carbon and respiration as well as the ratio of fungal to bacterial biomass varied along the experimental precipitation gradient and peaked under the +40% precipitation treatment. The shifts in microbial community composition could be largely attributable to the changes in soil water and nutrient availability. The metabolic quotient increased (indicating reduced carbon use efficiency) with increasing precipitation due to the leaching of dissolved organic carbon. The relative contributions of microbial respiration to soil and ecosystem respiration increased with increasing precipitation, suggesting that heterotrophic respiration will be more sensitive than autotrophic respiration if precipitation increases in the temperate steppe as predicted under future climate-change scenarios.

As results of increasing emission of greenhouse gases into the atmosphere, the Earth’s surface temperature has increased by 0.85 °C since 1880 and is predicted to increase by 1.0 to 3.7 °C by the end of this century^1^. Climatic warming alters precipitation amount and distribution by increasing the water-holding capacity of the atmosphere, enhancing the evaporation rate, and disrupting air circulation patterns^2^,^3^, leading to intensified intra- and inter-annual variations in precipitation amount in recent years^4^,^5^,^6^. However, while the enrichment of atmospheric greenhouse gases and climate warming is a global phenomenon, changes in precipitation patterns differ among regions. It has been predicted that annual mean precipitation will increase in high and many mid-latitude wet regions but will decrease in many mid-latitude and subtropical dry regions by the end of this century^1^,^7^. A better understanding of the effects of increased and decreased precipitation on the structure and function of terrestrial ecosystems is critical for predicting how ecological services will change under future climate-change scenarios.

Microorganisms play key roles in soil biogeochemical processes, including organic matter decomposition and nutrient mineralization^8^,^9^. Microbial abundance, community composition, and activity are directly affected by soil abiotic factors such as water and nutrient availability^10^,^11^,^12^, and indirectly affected by plant biomass and diversity^13^,^14^,^15^. Fungi and bacteria are the two major components of microbial decomposer communities. Fungi are typically considered more drought-tolerant than bacteria because fungal hyphae can transfer moisture from water-filled micropores to drained pores whereas bacteria require water films for motility and substrate diffusion^16^,^17^,^18^. However, two previous studies suggest that increases in water availability can stimulate fungal biomass or the ratio of fungi to bacteria in continuously moist soils due to more recalcitrant carbon inputs from plants^19^,^20^. More evidences concerning fungal and bacterial community composition under intensified precipitation variability are needed for understanding general response patterns of soil microorganisms.

Given the species- or functional group-specific sensitivity to water availability, shifts in microbial composition could lead to changes in microbial-associated processes and consequent changes in ecosystem functions. Heterotrophic respiration via soil microorganisms (microbial respiration) can account for 10–90% of the CO_2_ efflux from soils and substantially affect the atmospheric CO_2_ concentration^21^,^22^. Increasing precipitation can stimulate microbial respiration by increasing extracellular enzyme activities^18^,^23^ and the availability of substrates^24^,^25^. Heterotrophic respiration, however, can be suppressed under extremely moist conditions^26^,^27^. Accurate determination of heterotrophic respiration responses to precipitation variation could provide parameter estimates required for model simulations concerning future atmospheric CO_2_ concentrations^28^.

Arid and semiarid steppes are water-limited and particularly sensitive to altered precipitation regimes^29^. To investigate precipitation effects on microbial community and respiration, a field experiment with seven levels of precipitation manipulation (i.e., ambient precipitation as a control, and ±20%, ±40%, and ±60% of ambient precipitation) that cover the natural range in precipitation variation has been conducted in a semiarid temperate steppe in northern China since 2010. Given the differences of microbial tolerability and nutrients availability along precipitation gradient, we hypothesis that: 1) Relative bacterial and fungal dominance could be altered by precipitation variations. 2) Microbial respiration could be enhanced by increasing precipitation. However, this positive relationship may not be persistent when water no more limit. 3) Shifts in microbial composition could lead to changes in microbial respiration.

## Results

### Soil moisture and temperature

**Figure 1:**
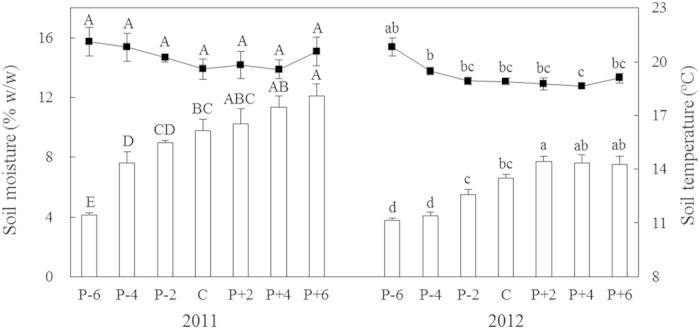
Soil moisture (bars) and temperature (lines) along the precipitation gradient on August 15^th^ of 2011 and 2012. Values are means ± S.E., n = 6. P-6, P-4, P-2, C, P + 2, P + 4, and P + 6 represent −60%, −40%, −20%, control (ambient), +20%, +40%, and +60% precipitation, respectively. Means with the same uppercase letter for 2011 or lowercase letter for 2012 are not significantly different (*P* > 0.05).

Soil moisture (SM) varied with year (*P* < 0.001; Table 1). Mean SM in 2011 (9.2%) was higher than that in 2012 (6.1%; Fig. 1). Precipitation treatment significantly affected SM (*P* < 0.001; Table 1). Across the two years, SM increased gradually with increasing precipitation and reached a maximum of 9.8% under the +60% precipitation treatment. Soil temperature (ST) at the 10 cm depth showed strongly inter-annual variability (*P* < 0.001; Table 1). Mean ST in 2011 (20.2 °C) was higher than that in 2012 (19.2 °C; Fig. 1). Precipitation significantly influenced ST (*P* < 0.01; Table 1). ST declined along the precipitation gradient and reached the minimum of 19.6 and 18.6 °C under the +40% precipitation treatment in 2011 and 2012, respectively (Fig. 1).

### Soil microbial biomass, community composition, and respiration

**Figure 2:**
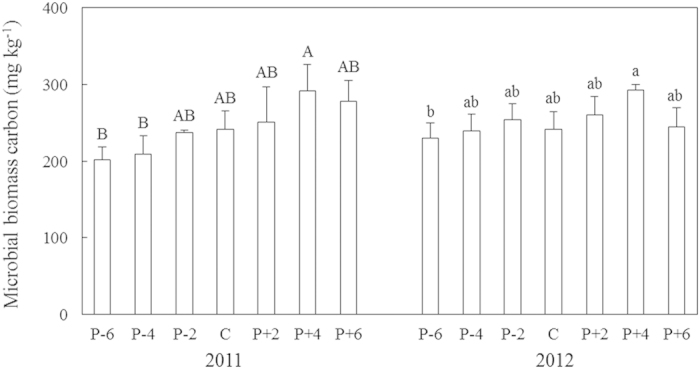
Microbial biomass carbon (MBC) in 2011 and 2012 along the precipitation gradient. Values are means ± S.E., n = 6. P-6, P-4, P-2, C, P + 2, P + 4, and P + 6 represent −60%, −40%, −20%, control (ambient), +20%, +40%, and +60% precipitation, respectively. Means with the same uppercase letter for 2011 or lowercase letter for 2012 are not significantly different (*P* > 0.05).

No inter-annual variation in microbial biomass carbon (MBC) was detected (*P* > 0.05). Precipitation treatment marginally impacted MBC (*P* < 0.10; Table 1). MBC tended to increase along the precipitation gradient and peaked under the +40% precipitation treatment in both 2011 and 2012 (291.5 and 292.5 mg kg^−1^; Fig. 2).

**Figure 3:**
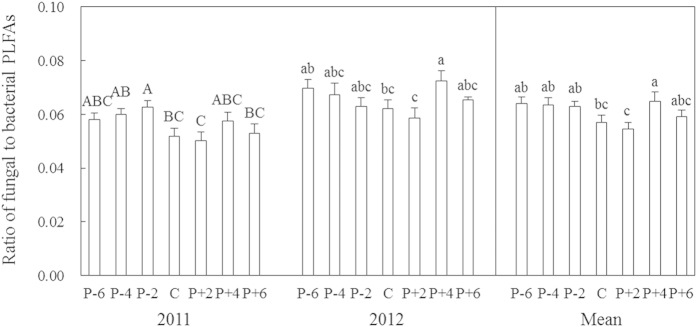
The ratio of fungal to bacterial PLFAs in 2011 and 2012 and their two-year averages along the precipitation gradient. Values are means ± S.E., n = 6 for each year and 12 for both years. P-6, P-4, P-2, C, P + 2, P + 4, and P + 6 represent −60%, −40%, −20%, control (ambient), +20%, +40%, and +60% precipitation, respectively. Means with the same letter are not significantly different (*P* > 0.05).

Year and precipitation did not influence total microbial phospholipid fatty acids (PLFAs; *P* > 0.05). However, significant effects of year and precipitation on the relative mole percentage of fungal PLFAs (*P* < 0.001, *P* < 0.05) and the ratio of fungal to bacterial PLFAs (F/B; *P* < 0.001, *P* < 0.01) were found (Table 1). Mean F/B in 2011 (0.056) was lower than that in 2012 (0.066; Fig. 3). Across the two years, F/B declined along the precipitation gradient, reached a minimum of 0.055 under the +20% precipitation treatment, and then increased at the two highest water addition levels (Fig. 3). Inter-annual variations in the relative mole percentage of Gram-negative bacterial PLFAs (GN%; *P* < 0.001), the relative mole percentage of Gram-positive bacterial PLFAs (GP%; *P* < 0.05), and the ratio of Gram-negative to Gram-positive bacterial PLFAs (GN/GP; *P* < 0.001) were also observed (Table 1). The absolute value of GN% in 2012 (23.3%) was lower than that in 2011 (25.1%) whereas GP% (29.9%) was greater in 2012 than 2011 (29.1%), which resulted in lower GN/GP in 2012.

**Figure 4:**
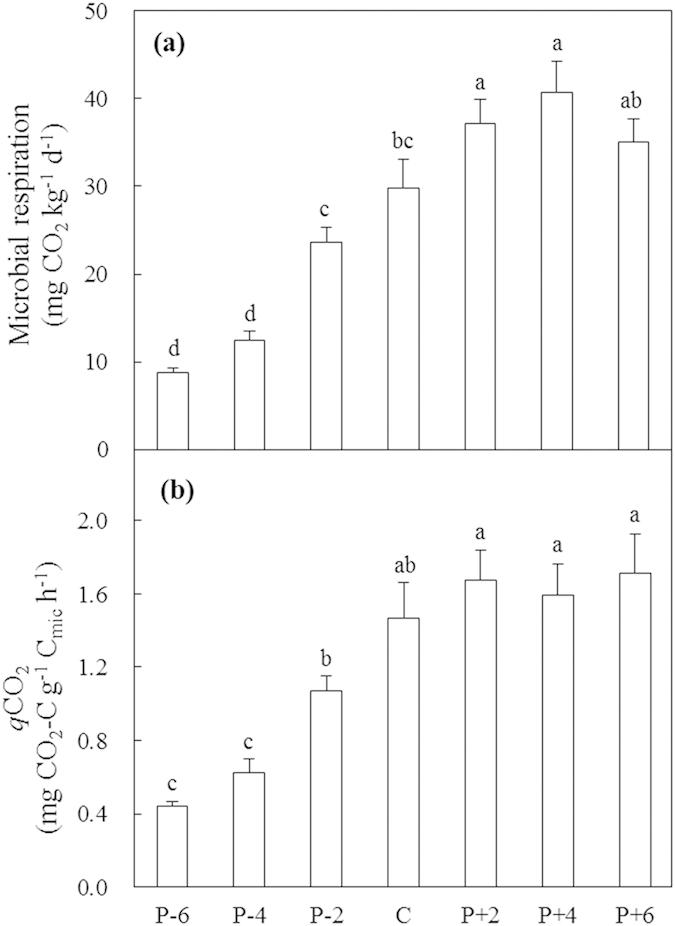
Microbial respiration (**a**) and metabolic quotient (*q*CO_2_, (**b**) in 2012 along the precipitation gradient. Values are means ± S.E., n = 6. P-6, P-4, P-2, C, P + 2, P + 4, and P + 6 represent −60%, −40%, −20%, control (ambient), +20%, +40%, and +60% precipitation, respectively. Means with the same letter are not significantly different (*P* > 0.05).

The effects of precipitation on microbial respiration (MR; *P* < 0.001) and metabolic quotient (*q*CO_2_; *P* < 0.001) were significant in 2012 (Table 1). MR increased from 8.7 mg CO_2_ kg^−1^ d^−1^ under the −60% precipitation treatment to 40.6 mg CO_2_ kg^−1^ d^−1^ under the +40% precipitation treatment, and then reduced under the +60% precipitation treatment (Fig. 4a). Decreased precipitation, on average, suppressed mean MR by 49.9% whereas increased precipitation stimulated MR by 26.1% (Fig. 4a). The *q*CO_2_ increased from 0.4 mg CO_2_-C g^−1^ C_mic_ h^−1^ under the −60% precipitation treatment to 1.7 mg CO_2_-C g^−1^ C_mic_ h^−1^ under the +20% precipitation treatment, and remained constant with further increases in precipitation (Fig. 4b). On average, decreased precipitation reduced mean *q*CO_2_ by 51.7% whereas increased precipitation enhanced *q*CO_2_ by 12.9% (Fig. 4b).

### Relationships of soil microbial parameters with biotic and abiotic factors

**Figure 5:**
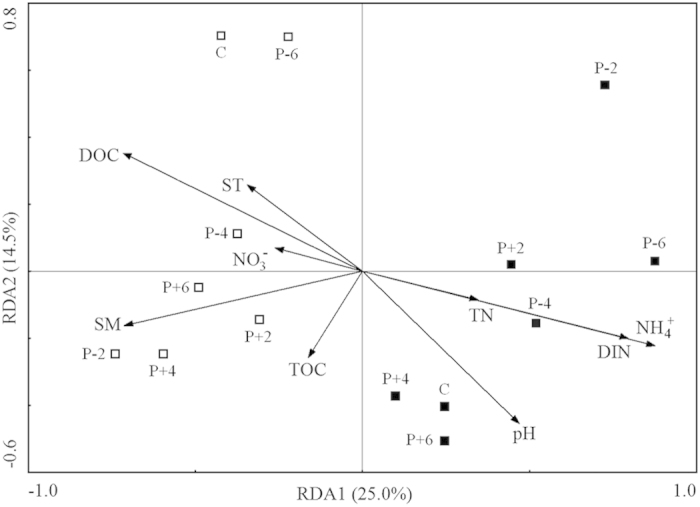
Redundancy analysis (RDA) of the associations of soil microbial community composition (as indicated by PLFAs) with environmental factors. P-6, P-4, P-2, C, P + 2, P + 4, and P + 6 represent −60%, −40%, −20%, control (ambient), +20%, +40%, and +60% precipitation, respectively. “Hollow squares” and “solid squares” represent 2011 and 2012, respectively. SM, soil moisture; ST, soil temperature; DOC, dissolved organic C; DIN, dissolved inorganic N; TOC, total organic C; TN, total N.

The result of Redundancy Analyses (RDA) showed that the variation of PLFA data was explained 25.0% and 14.5% by RDA1 and RDA2 axes, respectively. Microbial community composition in 2012 was set apart from 2011 along the RDA1, showing a substantial difference between the two years (Fig. 5). RDA1 was highly related to NH_4_^+^-N, dissolved inorganic N, dissolved organic C, and SM with correlation coefficient of 0.89, 0.81, −0.73, and −0.73, respectively (Fig. 5). A negative correlation was detected between the PLFA index of physiological stress and SM (r^2^ = 0.18; *P* < 0.001).

**Figure 6:**
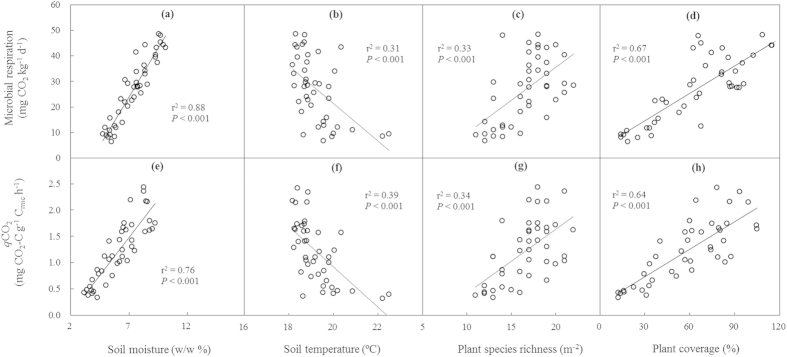
Dependence of microbial respiration and metabolic quotient (*q*CO_2_) on soil moisture, soil temperature, plant species richness, and plant coverage across all plots and both years.

Across the two years and all the plots, MR linearly increased with SM (Fig. 6a), plant species richness (Fig. 6c), and plant coverage (Fig. 6d), but declined with ST (Fig. 6b). Metabolic quotient showed positive correlations with SM (Fig. 6e), plant species richness (Fig. 6g), and plant coverage (Fig. 6h), but a negative correlation with ST (Fig. 6f). Multiple stepwise regressions showed that SM (partial r^2^ = 0.58; *P* < 0.001) and plant coverage (partial r^2^ = 0.05; *P* < 0.05) together explained 63% of the variations in MR. SM (partial r^2^ = 0.73; *P* < 0.001) and dissolved organic carbon (partial r^2^ = 0.05; *P* < 0.01) together accounted for 78% of the variations in *q*CO_2_.

### Contributions of microbial respiration to soil and ecosystem respiration

**Figure 7:**
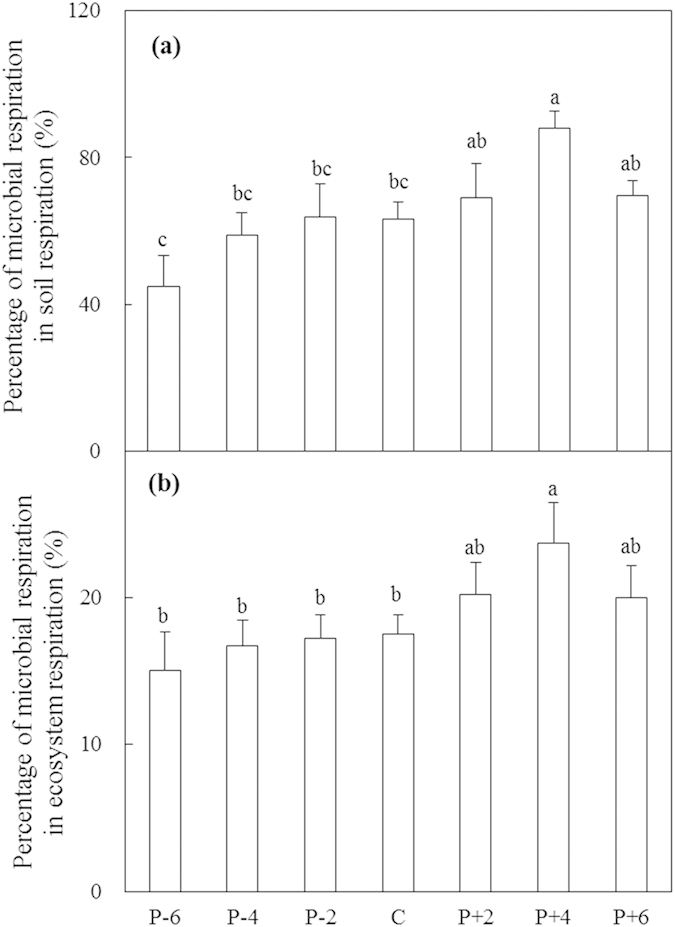
Relative contributions of microbial respiration to soil respiration (**a**) and to ecosystem respiration (**b**) in 2012 along the precipitation gradient. Values are means ± S.E., n = 6. P-6, P-4, P-2, C, P + 2, P + 4, and P + 6 represent −60%, −40%, −20%, control (ambient), +20%, +40%, and +60% precipitation, respectively. In each panel, means with the same letter are not significantly different (*P* > 0.05).

The effect of precipitation on the relative contributions of MR to soil respiration was significant in 2012 (*P* < 0.01; Fig. 7a). On average, decreased precipitation (−20%, −40%, and −60% precipitation) reduced the relative contributions of MR to soil respiration by 7.3% whereas increased precipitation (+20%, +40%, and +60% precipitation) increased the relative contributions of MR to soil respiration by 12.3% (absolute change; Fig. 7a). No effect of precipitation on the relative contribution of MR to ecosystem respiration was found (*P* > 0.05). However, mean contribution of MR to ecosystem respiration was higher under increased (21.3%) than decreased precipitation (16.3%; Fig. 7b).

## Discussion

In the present study, decreased precipitation increased the relative mole percentage of fungal PLFAs and F/B PLFAs, suggesting that the fungi in these communities are more drought-tolerant than the bacteria (Table 1; Fig. 3). This is consistent with findings of some previous studies^16^,^30^. Fungi can obtain water resources from water-filled micropores relatively easily through hyphal extension^31^. Although the increased F/B under the +40% precipitation treatment appears to be inconsistent with this general pattern, the discrepancy may be attributed to the greater production of roots and of recalcitrant materials under moist conditions^32^. These findings support our first hypothesis. Aboveground net primary production peaked under the +40% precipitation treatment (data not shown), which probably increased soil moisture loss via plant transpiration. Soil moisture was indeed lower under the +40% precipitation than +20% precipitation treatments according to long-term observation data (2010–2012). This could help to explain the higher F/B under the +40% precipitation treatment.

Microbial community composition also differed between years due to the differences in water and nutrient availability (SM, dissolved inorganic N, and organic C) in the absence of precipitation manipulation (RDA; Fig. 5). Microbial communities showed stronger responses to the between-year differences in rainfall than to precipitation manipulation^9^. Although total rainfall during the growing season of 2011 and 2012 was almost the same (279 and 298 mm, respectively), rainfall in August (the sampling period) of 2012 was only 15 mm but was 31 mm in August of 2011. The absolute difference in natural precipitation in August between the two years was greater than the difference caused by precipitation manipulations. Between-year differences in the microbial community could also reflect the relative abundances of GN and GP (Table 1). GP possess a stronger cell wall and a potentially more advanced osmoregulatory strategy than GN, suggesting that GP should be more tolerant of the drought conditions that occurred in 2012^10^,^33^.

In this study, MBC, MR, and *q*CO_2_ were positively related to SM, suggesting that water availability is an important abiotic factor regulating microbial properties^31^,^34^. Enhancement of soil water availability can increase MBC and MR by supporting the diffusion of dissolved organic C and inorganic N in water films and by stimulating extracellular enzyme activities^23^,^35^. In addition, the significant positive correlations of MR and *q*CO_2_ with plant species richness and coverage indicate that plants could have indirectly influenced microbial activity (Fig. 6c,d,g,h). Higher plant species richness and coverage may increase the variety and quantity of plant-produced organic C inputs into soil, increasing microbial functional diversity^36^,^37^. The negative correlation of MR with ST in this study is inconsistent with most previous studies. With the increase in SM, ST decreases due to evaporation. In arid and semiarid ecosystems, moisture is the primary limiting factor and more important than temperature in regulating microbial processes^34^. The negative correlation may not represent a direct effect of temperature on MR.

The decline in the index of physiological stress indicates that environmental conditions get better with increasing precipitation. Both MBC and MR increased along the precipitation gradient and reached maximal values under the +40% precipitation treatment, indicating that water no longer limits microbial growth in that treatment (Figs 2 and 4a). The results are consistent with the second hypothesis. The addition of more water, may reduce air movement through soil pores and thus limit O_2_ diffusion into soil and CO_2_ release from soil^38^. The metabolic quotient reached its maximum (lowest microbial C use efficiency) and entered a stable stage under the increased precipitation treatments, indicating that microorganisms consume a higher proportion C for maintenance than for biosynthesis under moist rather than dry conditions^39^,^40^ (Fig. 4b). This uncommon pattern of a higher metabolic quotient under a less stressful condition could be interpreted in two ways. On the one hand, because not all microorganisms are active or growing, a larger proportion of total biomass that is metabolically active would produce a higher metabolic quotient^41^. On the other hand, the sandy soil at the study site could enhance the leaching of dissolved organic C. A reduction in dissolved organic C with increased precipitation may lead to lower C use efficiency. Microbial communities are important components of terrestrial ecosystems, and alterations in their functional group composition under climate change could affect microbial and ecosystem functions. However, no dependence of MR and *q*CO_2_ on microbial community was found in this study. The third hypothesis could not be verified by direct evidence. Although a few studies have reported that MR was significantly correlated with F/B or GN/GP^42^,^43^, the general patterns of associations of microbial structure and function are not clear. Microbial function processes are complex and closely related to environmental conditions and microbial physiological status. Environmental conditions, such as water and nutrients availabilities, may play greater roles in regulating microbial respiration than community changes. Microbial respiration is a main component of soil and ecosystem respiration, changes in heterotrophic respiration could affect atmosphere CO_2_ concentration and result in positive feedback to climate change. In this study, microbial respiration measured based on topsoil of 0–10 cm in laboratory is not representative of natural heterotrophic respiration in whole soil layers. The relative contribution of microbial respiration is not actual, however, it may reflect at least in part. The relative contributions of microbial respiration to soil and ecosystem respiration increased under the increased precipitation treatments, suggesting that increased precipitation affects heterotrophic respiration more strongly than autotrophic respiration in the semiarid steppe of northern China. Soil C release is likely to increase in semiarid temperate steppe of northern China under future precipitation increase scenarios. Our findings facilitate the mechanistic understanding of how semiarid temperate steppe ecosystem may respond to changing precipitation regimes and extreme precipitation events.

## Methods

### Site description and experimental design

The study was conducted in a semiarid temperate steppe located in Duolun County (42°02′ N, 116°17′ E, 1324 m a.s.l.), Inner Mongolia, China. Over the past 60 years, the mean annual precipitation is 379 mm, the mean annual temperature is 2.1 °C, and the minimum and maximum temperatures range from −17.5 °C in January to 18.9 °C in July. The sandy soil is classified as Haplic Calcisols according to the FAO (Food and Agriculture Organization of the United Nations) and has a mean bulk density of 1.31 g cm^−3^. Plant communities at our experimental site are dominated by *Stipa krylovii*, *Artemisia frigida*, *Potentilla acaulis*, *Cleistogenes squarrosa*, and *Agropyron cristatum*. The average plant height is 20–40 cm.

The experiment used a randomized complete block design with seven treatments, including: −60%, −40%, −20% of ambient precipitation; ambient precipitation as a control; and +20%, +40%, and +60% of ambient precipitation. Each treatment was replicated six times, and each replicate plot was 4 × 4 m^2^. The precipitation treatments have been applied from June to September of each year beginning in 2010. The range of precipitation values achieved in the experiment was larger than the natural inter-annual range (−34.8% to +34.8%) and the range from June to September (−54.2% to +41.9%) over the past 60 years. For decreased precipitation plots, V-shaped rainout shelters, which were made with different numbers of transparent plexiglass bands, were used to intercept different amounts of incoming rainfall^44^. For increased precipitation plots, the appropriate volume of rain was added manually after each rainfall event. To reduce variation in sunlight radiation, flat plexiglass bands were installed in the control and increased-precipitation plots. Precipitation frequency and timing were not changed by our treatments.

### Soil sampling and analysis

Soil samples were collected from all 42 plots on August 15^th^ of 2011 and 2012. In each plot, four soil cores (0–10 cm depth, 5 cm diameter) were randomly taken using an auger and were mixed to obtain one composite sample per plot. After visible roots and stones were removed by hand and the soil was passed through a 2-mm sieve, subsamples were placed in an icebox and transported to the laboratory for microbial analysis. Other subsamples were air-dried for chemical analysis.

Gravimetric soil moisture was measured by the oven-drying method. Soil temperature at 10 cm depth was determined using a thermocouple probe connected to an Automated Soil CO_2_ Flux System (LI-8100, Li-Cor, Lincoln, NE, USA). Dissolved inorganic nitrogen (NH_4_^+^-N, and NO_3_^−^-N) was extracted from 10 g of fresh soil with 50 ml of 2 M KCl, and was measured with a Discrete Auto Analyzer (SmartChem 200, WestCo Scientific Instruments Inc., Italy). Soil pH was determined in a soil water mixture of 1:2.5 ratio (w/v) with a pH meter (Sartorius Basic PH Meter PB-10, Göttingen, Germany). Soil total organic carbon and total nitrogen were measured using the solid combustion method with an Elemental Analyzer (Elementar vario MACRO CUBE, Elementar Co., Hanau, Germany).

Soil microbial biomass carbon was measured by the fumigation-extraction method^45^. Briefly, moist samples (15 g dry weight equivalent) were fumigated with ethanol-free CHCl_3_ for 24 h at 25 °C. The fumigated and unfumigated subsamples were then extracted after being shaken for 30 min in 60 ml of 0.5 M K_2_SO_4_. The extracts were filtered and frozen at −20 °C before analysis. Microbial biomass carbon was measured with a Total Organic Carbon Analyzer (Elementar vario TOC, Elementar Co., Germany) and was calculated as the difference in extractable organic carbon content between the fumigated and control subsamples using a conversion factor of 0.45^46^. The organic carbon in unfumigated soil extracts was used as dissolved organic carbon. Microbial respiration was measured based on alkali absorption of CO_2_ at 25 °C for 14 days followed by titration of the residual OH^−^ with a standardized HCl solution^47^. In brief, a fresh soil sample of 20 g was placed evenly on the bottom of a 500-ml glass flask with a connecting tube. A 5-ml volume of 0.05 M NaOH solution was then injected into the connecting tube to absorb the CO_2_ released from the soil in the flask. The glass flasks were incubated at 25 °C in the dark for 14 days. Microbial respiration in 2011 was not measured due to the lack of fresh samples. The metabolic quotient was calculated as microbial respiration divided by microbial biomass carbon^48^. Higher metabolic quotient values indicate a lower efficiency of soil microorganisms in utilizing organic carbon, i.e., higher values indicate that the microorganisms must spend more energy on maintenance. To calculate the relative contributions of microbial respiration to soil and ecosystem respiration, unit of microbial respiration was converted to the same unit of soil and ecosystem respiration according to soil bulk density as follow:


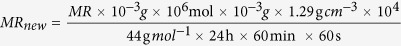


Phospholipid fatty acid (PLFA) analysis was used to evaluate microbial community composition^49^. In brief, PLFAs were extracted from fresh soil (8 g dry weight equivalent) with a single-phase mixture (1:2:0.8 v/v/v) containing chloroform, methanol, and phosphate buffer. The lipids were separated into neutral lipids, glycolipids, and phospholipids on a silicic acid column. The phospholipids were then subjected to a mild alkaline methanolysis and were dissolved in appropriate amounts of hexane containing 19:0 methyl esters as the internal standard. Finally, the samples were analyzed using a Gas Chromatograph (Agilent 7890 A, Agilent technologies, USA) and a Microbial Identification System (MIDI Inc., USA). The nomenclature of fatty acids is X:YωZ^50^, where X and Y represent the number of C atoms and double bonds, respectively, Z represents the position of the first double bond or cyclopropane ring, and ω refers to the position counting from the methyl end of the molecule. Bacterial biomass was estimated by the summed concentration of the fatty acids 15:0 a, 15:0 i, 16:0 i, 16:1ω7, 16:1ω9, 17:0 a, 17:0 cy, 17:0 i, 18:1ω5, 18:1ω7, and 19:0 cy, and fungal biomass was determined by 18:2ω6^49^,^51^. Mono-unsaturated and cyclopropyl fatty acids were used as indicators of Gram-negative bacteria, and iso and anteiso branched fatty acids were used as indicators of Gram-positive bacteria. The ratio of bacterial fatty acid 19:0 cyclo ω8 to its metabolic precursor 18:1ω7 was considered an indicator of physiological stress^52^. Higher index of physiological stress means more severe environmental pressures.

### Soil and ecosystem respiration measurements

To measure soil respiration, two PVC collars (11 cm in internal diameter and 5 cm in height) were inserted 3 cm into the soil at two opposite corners in each plot. All living plants inside the soil collars were removed by hand at least one day prior to the measurements to exclude plant respiration. Soil respiration was measured using an Automated Soil CO_2_ Flux System (LI-8100, Li-Cor, Lincoln, NE, USA). Measurements were taken by putting the LI-8100 chamber on the PVC collars for 1–2 min. The values of two collars in each subplot were averaged as one replicate. Ecosystem C flux was measured with a transparent chamber (0.5 × 0.5 × 0.5 m^3^) attached to an infrared gas analyzer (LI-6400, Li-Cor, Lincoln, NE, USA). The chamber was placed and sealed on an aluminum frame (0.5 × 0.5 m^2^) inserted 3 cm into the soil and then covered with an opaque cloth. Because of elimination of light, the values of C flux represented ecosystem respiration. Both soil and ecosystem respiration were measured between 9:00 and 12:00 AM on August 15^th^ of 2011 and 2012.

### Plant measurements

One frame (1 × 1 m), i.e., a quadrat, with 100 equally distributed grids was placed above the canopy at the center of each plot and was used to measure plant species richness and coverage in mid-August 2011 and 2012. Species richness was recorded as the number of plant species in the quadrat. Coverage of all species, i.e., community coverage, was visually estimated in the quadrat.

### Statistical analysis

The effects of year, precipitation (i.e., precipitation treatments), and their potential interactions on soil parameters, microbial biomass carbon, and the relative mole percentage of main microbial group PLFAs were analyzed using two-way ANOVAs. Multiple comparisons were used to examine the effects of the seven precipitation treatments on microbial respiration, metabolic quotient, and the above variables in each year. Simple and multiple linear stepwise regressions were performed to assess the relationships of microbial biomass carbon, microbial respiration, and metabolic quotient with biotic (plant species richness and coverage) and abiotic factors (soil moisture, soil temperature, dissolved organic carbon, and dissolved inorganic nitrogen). These ANOVAs and regressions were performed using SPSS 17.0 (SPSS Inc., Chicago, USA). Redundancy Analyses were performed using Canoco 4.5 (Ithaca, NY, USA) to evaluate the relationship between microbial community composition and soil controlling factors (soil moisture, soil temperature, dissolved organic carbon, dissolved inorganic nitrogen, NH_4_^+^-N, NO_3_^−^-N, total organic carbon, total nitrogen, and pH). PLFA data were calculated as mole percent and were log transformed before analyses.

## Additional Information

**How to cite this article**: Zhao, C. *et al.* Soil microbial community composition and respiration along an experimental precipitation gradient in a semiarid steppe. *Sci. Rep.* **6**, 24317; doi: 10.1038/srep24317 (2016).

## Figures and Tables

**Table 1:** Results (*F*-values) of two-way ANOVAs for the effects of year (Y), precipitation treatment (P), and their interactions (Y × P) on soil moisture (SM), soil temperature (ST), microbial biomass carbon (MBC), the mole percentage of fungal PLFAs (F%), the ratio of fungal to bacterial PLFAs (F/B), the mole percentage of Gram-negative bacterial PLFAs (GN%), the mole percentage of Gram-positive bacterial PLFAs (GP%), and the ratio of Gram-negative to Gram-positive bacterial PLFAs (GN/GP) over the two years of the study, and of one-way ANOVAs for the effects of precipitation treatment (P) on microbial respiration (MR) and metabolic quotient (*q*CO_2_) in 2012.

Source ofvariation	SM	ST	MBC	F%	F/B	GN%	GP%	GN/GP	MR	*q*CO_2_
Y	101.53***	13.78***	0.33	16.11***	30.54***	14.38***	6.37*	28.14***		
P	28.34***	3.19**	2.08^	2.90*	3.28**	0.38	2.12	0.92	25.54***	12.79***
Y × P	2.86*	0.33	0.37	1.72	1.15	1.34	1.21	1.57		

Significance levels: ^^^*P* < 0.10, *P < 0.05, **P < 0.01, ***P < 0.001.
